# Sesamol Ameliorates Renal Injury-Mediated Atherosclerosis Via Inhibition of Oxidative Stress/IKKα/p53

**DOI:** 10.3390/antiox10101519

**Published:** 2021-09-24

**Authors:** Jie-Sian Wang, Ping-Hsuan Tsai, Kuo-Feng Tseng, Fang-Yu Chen, Wen-Chin Yang, Ming-Yi Shen

**Affiliations:** 1 Graduate Institute of Biomedical Sciences, China Medical University, Taichung 40402, Taiwan; d29745@mail.cmuh.org.tw (J.-S.W.); u105010312@cmu.edu.tw (P.-H.T.); fyc0321@gmail.com (F.-Y.C.); 2 Division of Nephrology, Department of Internal Medicine, China Medical University Hospital, Taichung 40402, Taiwan; 3 Department of Biological Science and Technology, China Medical University, Taichung 40402, Taiwan; u107010409@cmu.edu.tw; 4 Agricultural Biotechnology Research Center, Academia Sinica, 128, Sec. 2, Academia Rd., Nankang, Taipei 115, Taiwan; wcyang@gate.sinica.edu.tw; 5 Department of Medical Research, China Medical University Hospital, Taichung 40402, Taiwan; 6 Department of Nursing, Asia University, Taichung 41354, Taiwan

**Keywords:** atherosclerosis, apoptosis, chronic kidney disease, sesamol, reactive oxygen species

## Abstract

Patients with chronic kidney disease (CKD) are at an increased risk of premature death due to the development of cardiovascular disease (CVD) owing to atherosclerosis-mediated cardiovascular events. However, the mechanisms linking CKD and CVD are clear, and the current treatments for high-risk groups are limited. In this study, we aimed to examine the effects of sesamol, a natural compound extracted from sesame oil, on the development of atherosclerosis in a rodent CKD model, and reactive oxygen species-induced oxidative damage in an endothelial cell model. ApoE^–/–^ mice were subjected to 5/6 nephrectomy (5/6 Nx) and administered sesamol for 8 weeks. Compared with the sham group, the 5/6 Nx ApoE^–/–^ mice showed a significant increase in malondialdehyde levels and Oil Red O staining patterns, which significantly decreased following sesamol administration. Sesamol suppressed H_2_O_2_-induced expression of phospho-IKKα, p53, and caspase-3. Our results highlight the protective role of sesamol in renal injury-associated atherosclerosis and the pathological importance of oxidative stress burden in CKD–CVD interaction.

## 1. Introduction

Patients with chronic renal failure are at an increased risk of premature mortality, which is associated with an increased incidence of atherosclerotic cardiovascular complications [1]. Atherosclerosis and cardiovascular diseases (CVDs) occur due to the imbalances between the production and scavenging of free radicals during physiological processes, thereby leading to inflammation, apoptosis, and lipid, protein, or nucleic acid damage [2]. Oxidative stress is caused by excessive reactive oxygen species (ROS), including hydroxyl radicals and superoxide anions [2]. Chronic kidney disease (CKD) is associated with high levels of ROS generation [3]. ROS might contribute to renal injury progression and atherosclerosis pathogenesis in CKD. Hydrogen peroxide (H_2_O_2_) is a major ROS generated during ischemia–reperfusion injury and is produced in animal models of chronic brain disease [4]. H_2_O_2_ can induce oxidative stress in endothelial cells, causing cell dysfunction and apoptosis [5,6]. Endothelial cells have a fundamental role in various pathological and physiological processes. For instance, they can synthesize and release catecholamines in response to ischemia, highlighting their importance in controlling vascular homeostasis [7]. Therefore, interventions that inhibit ROS-induced endothelial cell damage can benefit patients with CKD and CVD [3,8]. Oxidative stress can be alleviated by antioxidant therapies using vitamin C, vitamin E, and other natural antioxidants.

There is a growing interest in the development of effective cardiovascular therapeutic drugs, especially those of natural origin, owing to their lack of adverse effects and effects on a variety of disease targets [9,10]. Sesamol (SM), a predominant active component of sesame seed oil, has been recognized as a health food, and has been used as a traditional medicine for several years [11]. It is a safe, non-toxic, organic compound that mediates anti-inflammatory effects via the downregulation of inflammatory marker transcription, such as cytokines, protein kinases, enzymes that promote inflammation, and redox status [12,13]. Sesamol also possesses other therapeutic properties, including anti-cancer [14], anti-aging [15], anti-atherosclerotic [11], hepatoprotective [16], and anti-acute renal injury [17] effects. Although recent studies have demonstrated that SM supplementation acts as a potent adjuvant to treat acute renal injury owing to its prophylactic protective effects [17] and vascular protective effects [18,19,20], the mechanisms by which SM protects against renal injury-associated atherosclerosis and protects endothelial cells from oxidative stress injury remain unclear. In this study, we aimed to explore the mechanisms by which SM exerts its therapeutic effects on atherosclerosis in a chronic renal failure model and its effect on endothelial functions to identify alternative therapeutic interventions for cardiovascular complications in patients with CKD.

## 2. Materials and Methods

### 2.1. Reagents, Antibodies, and Cells

**Figure 1:**
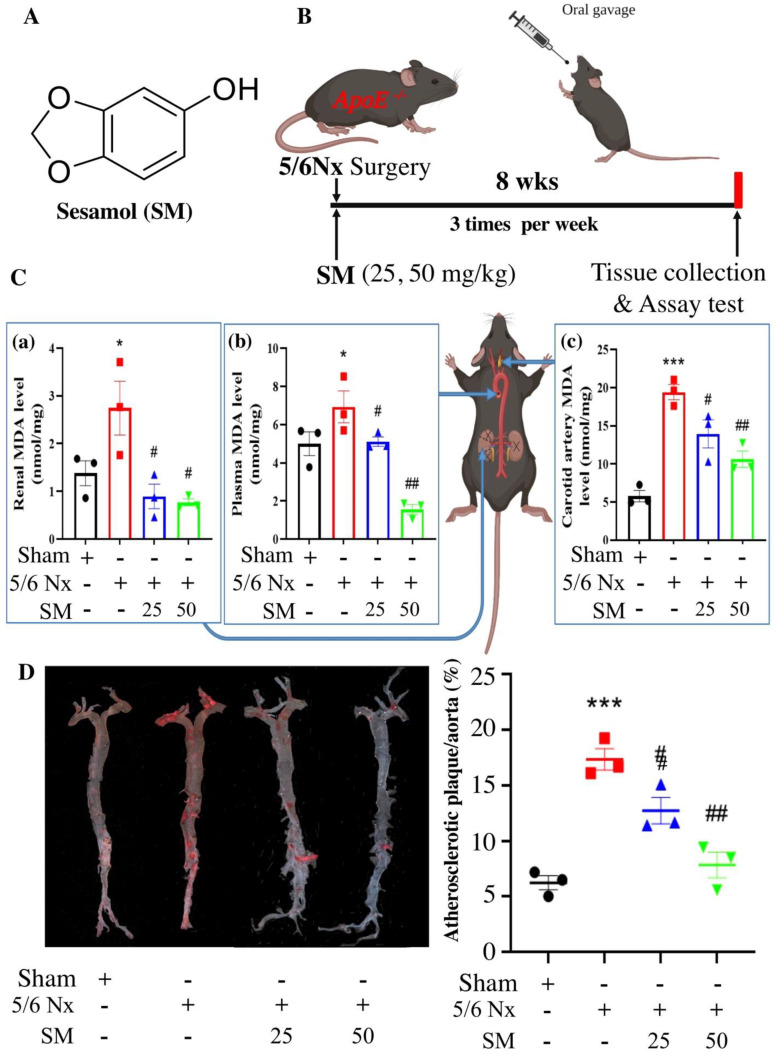
Sesamol (SM) reduces reactive oxygen species (ROS) levels and atherosclerosis in 5/6 Nx ApoE^–/–^ mice. (**A**) Structure of SM. (**B**) Schematic representation of the experimental mouse model. (**C**) Lipid peroxidation was assessed by measuring malondialdehyde (MDA) level. MDA level in (a) the kidney, (b) plasma, and (c) carotid artery in ApoE^–/–^ mice. (**D**) Representative aortas from mice in each group stained with Oil Red O and quantification of aortic root lesion sizes. Data are presented as mean ± SEM (*n* = 3 per group). * *p* < 0.05, *** *p* < 0.001 vs. sham group; # *p* < 0.05, ## *p* < 0.01 vs. 5/6 Nx group in ApoE^–/–^ mice.

SM (5-hydroxy-1,3-benzodioxole; purity: 99.9% by GC) (Figure 1A), dimethylsulfoxide (DMSO), H_2_O_2_, and 3-(4,5-di-methylthiazol-2-yl)2,5-diphenyltetrazolium bromide (MTT) were procured from Sigma (St. Louis, MO, USA). IKKα siRNA (si-IKKα) and control siRNA (si-Ctl) were bought from Santa Cruz Biotechnology (Santa Cruz, CA, USA). Human aortic endothelial cell line (HAEC), derived from a human heart aorta, was obtained from Promo Cell (Heidelberg, Germany).

### 2.2. Animal Model and Experimental Design

All mice experiments were approved by the China Medical University Institutional Animal Care and Use Committee, and were conducted in accordance with the Guide for the Care and Use of Laboratory Animals of the US National Institutes of Health (NIH Publication No. 85–23, revised 1996). Subtotal 5/6 nephrectomy (5/6 Nx) was performed on 10–12-week-old female ApoE^–/–^ mice under isoflurane inhalation anesthesia with total removal of the right kidney and selective infarction of approximately 2/3 of the left kidney by ligating two or three renal artery branches. Female ApoE^–/–^ mice were utilized because of an increased incidence of atherosclerosis in females compared to males [21]. The mice were fed a regular chow diet throughout the study. The mice received SM (25 and 50 mg/kg, SM25 and SM50, respectively) by oral gavage three times per week for 8 weeks after 5/6 Nx surgery. All mice were sacrificed via isoflurane inhalation followed by cervical dislocation [21] (Figure 1B).

### 2.3. Lipid Peroxidation Assay, Oil Red O Staining, and Histology Staining of Aortas

Experimental mice were anesthetized and euthanized via cervical dislocation, and the blood (to obtain plasma), carotid artery, aorta, and kidneys were collected. Lipid peroxidation was evaluated using a lipid peroxidation (MDA) assay kit (no. ab118970; Abcam, Cambridge, UK) according to the manufacturer’s instructions. Atherosclerotic plaques were visualized using an Oil Red O Staining kit (Sigma), and images were captured with a Canon EOS 70D digital camera (Tokyo, Japan). The aortic roots and carotid artery were fixed overnight with 4% paraformaldehyde, after which 0.5% Oil Red O solution (Sigma-Aldrich) was used to stain the aortic roots for 24 h. The stained aorta were spread on the blackboard, and a digital camera was used to shoot under the same shooting parameters and the same lighting conditions. Carotid arteries were embedded in paraffin and serially sliced into 3 µm-thick sections. Every third slide from the serial sections was stained with hematoxylin and eosin (H&E), and images were captured using a Leica DM750 (Wetzlar, Germany). Images were analyzed using Image J (Version 1.52a, NIH, Bethesda, MD, USA).

### 2.4. Measurement of Cell Viability

For the cell viability assessment, 4 × 10^3^ cells were seeded in 96-well plates. After incubation with SM (0.3–3 µM) or the control vehicle (0.1% DMSO) for 60 min, the cells were treated with or without H_2_O_2_ (100 µM) for 24 h. Thereafter, 20 µL of MTT solution (5 mg/mL) was added to each well, and the plate was incubated at 37 °C for 4 h. Next, the supernatant was replaced with DMSO. Then, the cells were oscillated. Finally, the absorbance of the sample at 570 nm was measured using a microplate reader (Infinite M1000, TECAN, Mechelen, Belgium). All data were normalized to those of the corresponding controls, and the percentage of control was calculated.

### 2.5. Measurement of Intracellular ROS Levels in Cells

Intracellular ROS levels were evaluated using a cellular ROS/superoxide detection assay kit (Abcam, Cambridge, UK) according to the manufacturer’s instructions. Briefly, the cells were seeded at a density of 1.0 × 10^4^ cells/well in 96-well plates, and they were allowed to attach for 24 h. Following this, the culture supernatant was removed, and cells were washed with phosphate-buffered saline (PBS). Next, the ROS-specific stain 2′,7′-dichlorofluorescein diacetate (DCFH-DA) was added, and cells were incubated in the dark for 30 min at 37 °C. Thereafter, the cells were washed twice with PBS. The cells were then observed via fluorescence microscopy, and intracellular ROS levels were measured using an Infinite M1000 microtiter plate reader (Tecan Group AG, Männedorf, Switzerland) (Ex = 488 nm, Em = 520 nm).

### 2.6. Apoptosis Assay

HAECs were incubated with SM (0.3–3 µM) or the control vehicle (0.1% DMSO) for 60 min, and they were then treated with or without H_2_O_2_ (100 µM) for 24 h. The treated cells were stained with 1 μM Hoechst 33,342 (Molecular Probes, Eugene, OR, USA) and calcein acetoxymethyl ester (calcein-AM) (Molecular Probes). Fluorescence imaging was performed with an Olympus IX70 inverted microscope (Tokyo, Japan), and apoptotic cells were quantified as previously described [11].

### 2.7. Protein Extraction and Western Blotting

Cells were seeded at a density of 2 × 10^5^ cells/well into 6-well plates, and they were allowed to attach for 24 h. Subsequently, the cells were treated with vehicle, H_2_O_2_ (100 µM for 24 h) alone, or H_2_O_2_ (100 µM for 24 h) after SM pretreatment (0.3–3 µM for 1 h before the addition of H_2_O_2_). Subsequently, to obtain protein extracts, the cells were lysed and homogenized in RIPA lysis buffer in the presence of protease inhibitors (Roche Applied Science, Penzberg, Germany). The bovine serum albumin protein assay kit (Pierce Biotechnology Inc., Waltham, MA, USA) was used to measure protein concentrations. Cell lysates (containing 20 μg of protein) were loaded onto a 12% sodium dodecyl sulfate (SDS) polyacrylamide gel, and were separated by SDS polyacrylamide gel electrophoresis. The separated proteins were transferred onto a Hybond-PVDF membrane (GE Healthcare Amersham, Buckinghamshire), and were then blocked with SuperBlock (Pierce Biotechnology Inc.). Non-specific sites were blocked with blocking buffer (500 mL of 0.1% PBST containing 5 g PVP and 1.25 g BSA) for 1 h. For immunoblotting, the membranes were incubated overnight with one of the following primary antibodies at 4 °C: anti-p-IKK (1:1000; Cell Signaling, Danvers, MA, USA), anti-p53 (1:1000; Cell Signaling), anti-cleaved caspase-3 (1:1000; CC3, Cell Signaling), and anti-β-actin (1:10,000; Sigma) in PBST overnight at 4 °C. Anti-rabbit horseradish peroxidase-conjugated immunoglobulin G (1:1000; DakoCytomation, Glostrup, Denmark) in PBST (1 h, 37 °C) was used as a secondary antibody. β-Actin was used to verify an equal loading of proteins in each lane. Protein expression was assessed using ECL reagents (Millipore, Billerica, MA, USA), and was quantified using quantity video densitometry (G-box Image System; Syngene, Frederick, MD, USA) [11].

### 2.8. Quantitative Real-Time PCR

After homogenization of mice carotid arteries, samples were frozen with NucleoZOL at −80 °C, and the total RNA was isolated and extracted according to the manufacturer’s instructions. The iScript cDNA Synthesis Kit (BioRad, Hercules, CA, USA) was used to synthesize cDNA. Finally, Q SYBR Green Supermix (BioRad) was used for real-time PCR. The primers are listed in Table 1.

### 2.9. Statistical Analysis

Data are presented as mean ± standard error of mean (SEM) of *n* experiments. Data were compared using one-way analysis of variance (ANOVA) or *t*-test, where appropriate. Results with *p* < 0.05 were considered statistically significant. GraphPad Prism 5 software (GraphPad Software Inc., La Jolla, CA, USA) was used for all statistical analyses.

## 3. Results

### 3.1. Effects of SM on ROS Levels and Atherosclerosis in 5/6 Nx ApoE^–/–^ KO Mice

**Figure 2:**
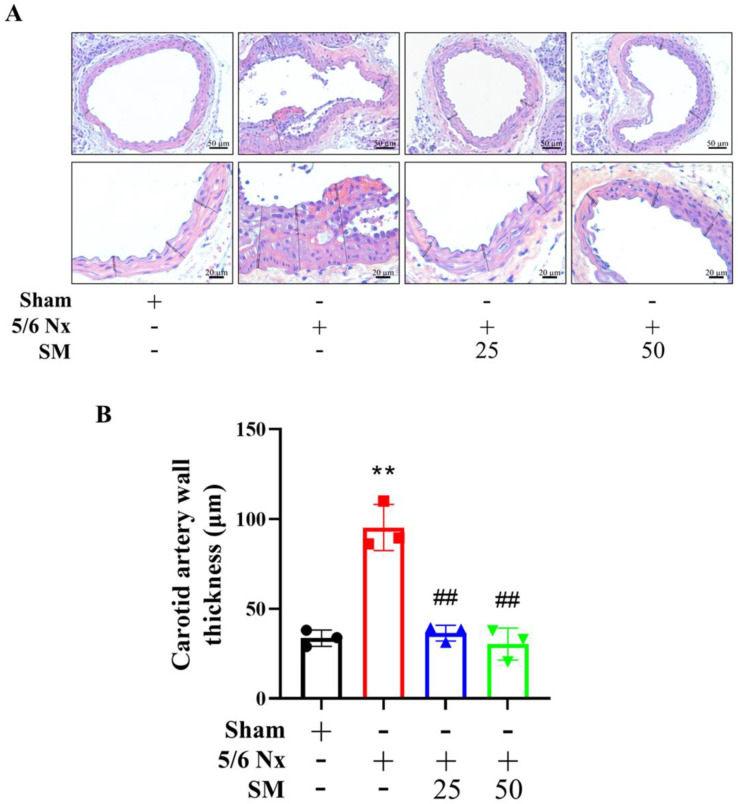
Hematoxylin and eosin staining shows the histopathology of atherosclerotic plaques. (**A**) Representative photomicrograph of ApoE^–/–^ mouse carotid artery (top panel, 200× and bottom panel, 400×). During the entire experiment, no obvious atherosclerotic lesions were found in the carotid artery of the sham group, and severe atherosclerosis was found in the carotid artery of the 5/6 Nx group. As the concentration of SM increased, the development of atherosclerotic lesions gradually decreased. (**B**) Wall thicknesses were quantified by unwitting researchers. Data are presented as mean ± SEM (*n* = 3 per group). ** *p* < 0.01 vs. sham group; ## *p* < 0.01 vs. 5/6 Nx group in ApoE^–/–^ mice.

SM25- and SM50-treated 5/6 Nx ApoE^–/–^ mice showed no difference in body weight or mortality rate. To investigate the effect of SM on oxidative stress, we evaluated the effect of SM supplementation on lipid peroxidation by measuring the MDA level in the kidney, plasma, and carotid artery. Compared with the sham group, 5/6 Nx ApoE^–/–^ mice (5/6 Nx) group showed increased MDA levels. However, the MDA level in the SM25 and SM50 groups decreased compared with those in the 5/6 Nx group (Figure 1C (a–c)). Additionally, to investigate whether SM is an effective therapeutic agent for CKD-mediated atherosclerosis, we assessed the effect of SM on atherosclerotic lesions in 5/6 Nx ApoE^–/–^ mice. Compared with the sham mice, the 5/6 Nx mice showed significantly increased atherosclerotic lesions as assessed by Oil Red O staining of the aorta (2.83-fold increase) (5/6 Nx vs. sham; *p* < 0.001). The aortas from the SM25 and SM50 group mice showed significantly reduced staining patterns when compared with those from the 5/6 Nx mice, with reductions of 16.3% and 58.8%, respectively (Figure 1D). Furthermore, the mice administered SM demonstrated a significant attenuation of carotid artery plaque formation, compared to the untreated mice. The plaque area was reduced after SM treatment in a dose-dependent manner (Figure 2A,B). At the 50 mg/kg dose, SM abrogated plaque formation, resulting in 70% reduction in plaque size compared to the untreated 5/6 Nx ApoE^–/–^ mice, as measured using morphometric analysis (Figure 2B, *p* < 0.01).

### 3.2. In Vitro Activity of SM on HAECs Injured Via H_2_O_2_ Treatment

**Figure 3:**
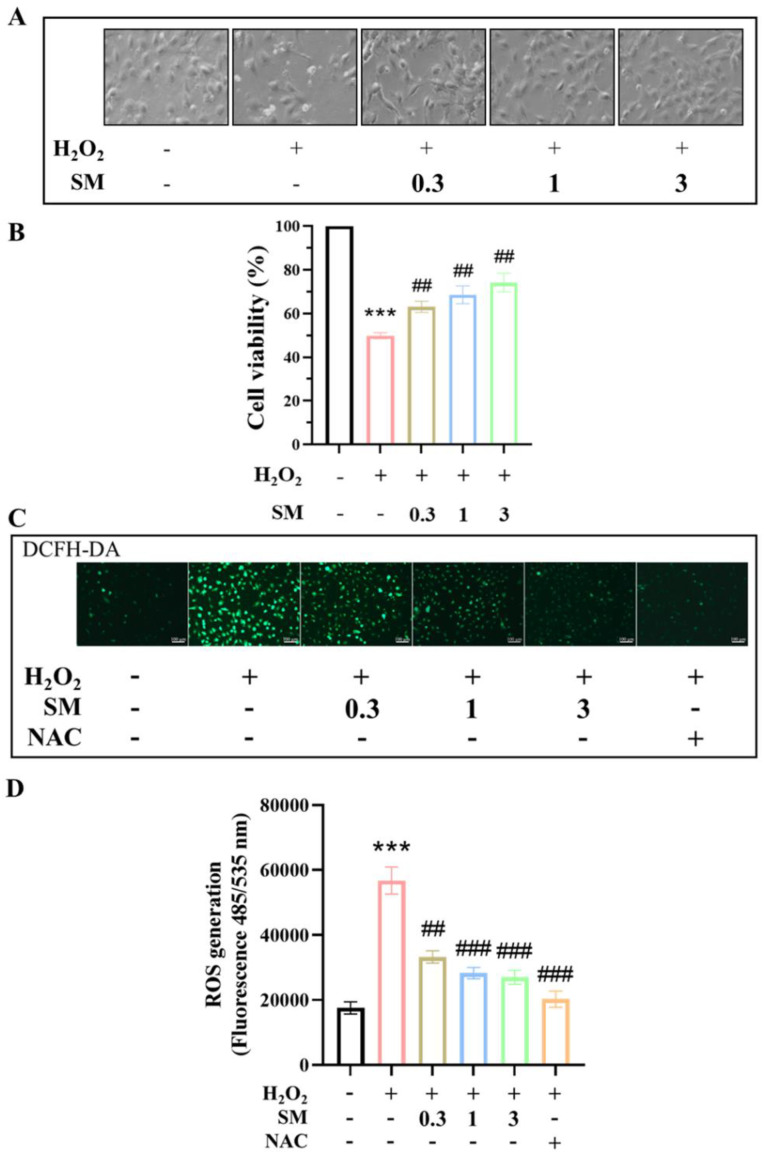
Effect of SM on the viability and intracellular ROS production of H_2_O_2_-treated human aortic endothelial cells (HAECs). (**A**) Representative images showing the effect of SM (0.3–3 µM) on HAEC morphological injury induced by H_2_O_2_ observed under a light microscope. (**B**) Following pretreatment with different concentrations of SM (0.3–3 μM) for 1 h, HAECs were treated with H_2_O_2_ (100 μM) for another 24 h; cell viability increased in a dose-dependent manner following SM treatment. Cell viability was determined using MTT assay. (**C**) A typical fluorescence image captured using a fluorescence microscope using 2′,7′-dichlorofluorescein diacetate to stain HAECs after H_2_O_2_ stimulation and SM incubation to observe intracellular ROS intensity; scale bar = 100 μm. (**D**) Cellular ROS/superoxide detection assay was performed. Data are presented as mean ± SEM. *p* values were determined using Student’s *t*-test. (*** *p* < 0.001 vs. control group, ## *p* < 0.01, ### *p* < 0.001 vs. H_2_O_2_ group). *N*-acetyl-l-cysteine (NAC, 3 mM).

We analyzed the effects of SM on H_2_O_2_-induced endothelial cell injury using cultured HAECs, and explored the underlying mechanisms. SM (3 μM) did not show any significant cytotoxic effect on HAECs, as determined using the MTT assay. Therefore, all subsequent experiments were conducted at doses less than 3 μM SM. To determine the protective effect of SM on H_2_O_2_-induced HAEC damage, cell survival was assessed using the MTT assay. The cells were pre-incubated with SM for 1 h and then with H_2_O_2_ (100 μM) for 24 h. HAEC viability significantly reduced after H_2_O_2_ treatment compared with control (*p* < 0.001, *n* = 4); however, pre-treatment with SM (0.3–3 μM) prevented the cell damage induced by 100 μM H_2_O_2_ (*p* < 0.01; *n* = 4; Figure 3A,B). These results suggest that SM might protect HAECs from ROS-related cellular damage. To further examine the protective effect of SM on H_2_O_2_-induced intracellular oxidative stress in HAECs, we measured intracellular ROS levels in H_2_O_2_-treated HAECs using DCFH-DA, an ROS-specific dye. Samples with higher concentration of ROS presented stronger green fluorescence intensity. The fluorescent intensity in H_2_O_2_-stimulated cells treated with SM (0.3, 1, and 3 μM) was weaker than that in H_2_O_2_-stimulated cells without any treatment (Figure 3C). Moreover, as shown in Figure 3D, H_2_O_2_ significantly increased intracellular ROS generation in HAECs (*p* < 0.001 vs. control, *n* = 3), and SM (0.3, 1, and 3 μM) treatment suppressed H_2_O_2_-induced ROS generation concentration-dependently (*p* < 0.001 at 3 μM vs. H_2_O_2_ alone; *n* = 3). Interestingly, the suppressive effect of 3 μM SM against H_2_O_2_-induced ROS generation was comparable to that of *N*-acetyl-l-cysteine (3 mM), a potent antioxidant.

### 3.3. Effects of SM on the Apoptosis of HAECs Exposed to H_2_O_2_

**Figure 4:**
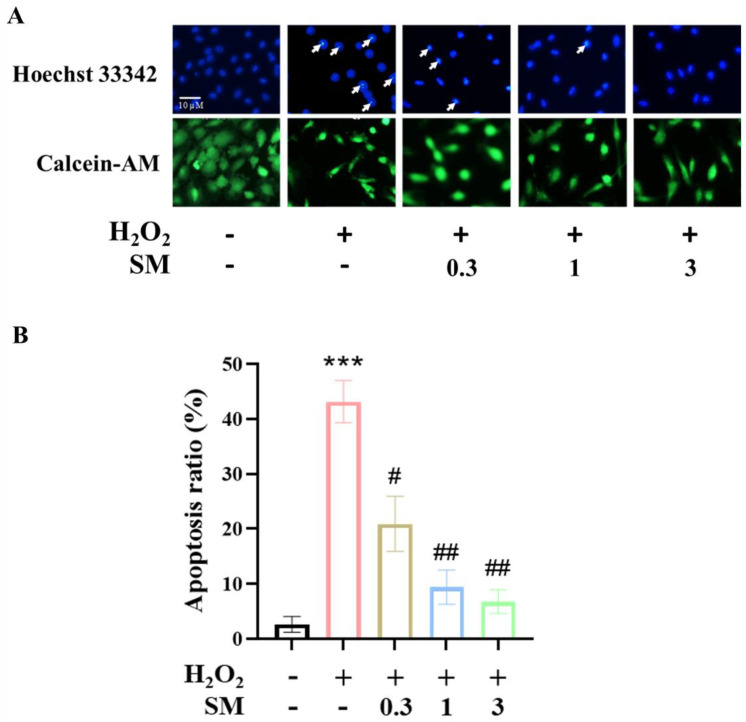
Protective effects of SM against H_2_O_2_-induced apoptosis in HAECs. HAECs were pretreated with SM (0.3–3 µM) for 30 min, and then incubated with or without 100 µM H_2_O_2_ for 24 h. (**A**) Epifluorescence microscopy images were obtained after staining with Hoechst 33,342 to assess nuclear morphology, and calcein-AM to evaluate membrane integrity. The presence of condensed and fragmented nuclei was considered an indicator of apoptosis (white arrow). (**B**) The mean percent cell apoptosis was evaluated in quadruplicate samples. The *p* values were determined using Student’s *t*-test. *** *p* < 0.001 vs. control group; # *p* < 0.05, ## *p* < 0.01 vs. H_2_O_2_-treated cells.

To elucidate whether SM suppresses H_2_O_2_-induced apoptosis in HAECs, cells were pretreated with increasing concentrations of SM (0.3–3 μM) and then exposed to H_2_O_2_ (100 μM). After 24 h, the cells were stained with Hoechst 33,342 and calcein-AM to assess nuclear morphology and membrane integrity. HAECs with condensed, fragmented nuclei were considered to be apoptotic. Fluorescence microscopy revealed that H_2_O_2_ alone induced cell apoptosis (*p* < 0.001), and pretreatment with SM suppressed H_2_O_2_-induced cell apoptosis dose dependently (0.3 μM, *p* < 0.05; 1 and 3 μM, *p* < 0.01 vs. H_2_O_2_-treated cells; Figure 4A,B).

### 3.4. Protective Effects of SM against H_2_O_2_-Induced Endothelial Cell Apoptosis Is Mediated via the IKKα Pathway

**Figure 5:**
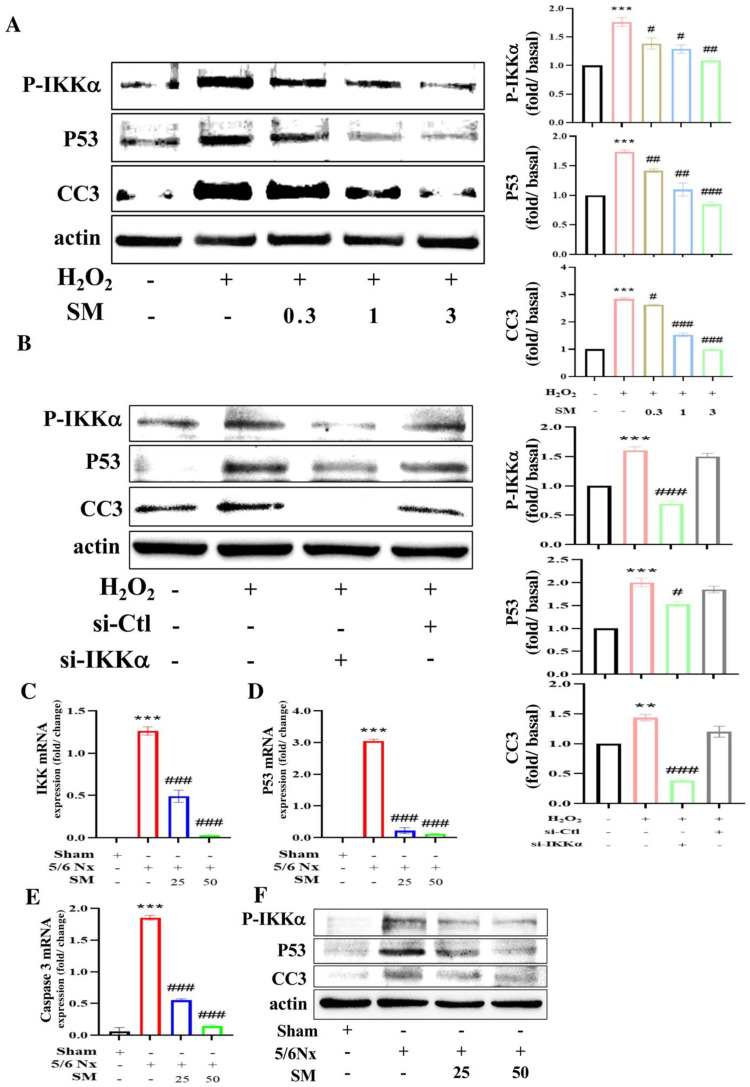
H_2_O_2_ significantly induces apoptosis in HAECs via the IKKα pathway, and pretreatment with SM blocks the activation of this pathway. (**A**) Western blot showing the effect of SM on the protein expression of P-IKKα, p53, and CC3 in H_2_O_2_-treated HAECs. (**B**) Western blotting showed that H_2_O_2_ induces apoptosis of HAECs via the IKKα pathway. In addition to H_2_O_2_, HAECs were treated with si-IKKα and si-Ctl (control). *p* values were determined using Student’s *t*-test. ** *p* < 0.01, *** *p* < 0.001 vs. control group; # *p* < 0.05, ## *p* < 0.01, and ### *p* < 0.001 vs. H_2_O_2_-treated cells. mRNA levels of (**C**) *IKKα*, (**D**) *p53*, and (**E**) *caspase-3* and (**F**) protein levels of P-IKKα, p53, and CC3 in the carotid arteries of SM-treated or untreated 5/6 Nx ApoE^–/–^ mice and sham were measured using quantitative real-time PCR, and they were normalized to the level of GAPDH mRNA. Data are presented as mean ± SEM (*n* = 3 per group). *** *p* < 0.001 vs. sham group; ### *p* < 0.001 vs. 5/6 Nx group in ApoE^–/–^ mice.

The levels of P-IKKα (a pro-inflammatory factor) and p53 (a pro-apoptotic factor) in H_2_O_2_-treated HAECs were determined by western blotting. As shown in Figure 5A, H_2_O_2_ treatment significantly increased the P-IKKα and p53 (*p* < 0.001 vs. control; *n* = 4) levels, whereas SM pretreatment significantly reduced the H_2_O_2_-mediated effects dose dependently (expression of P-IKKα, 0.3 µM and 1 µM SM, *p* < 0.05; 3 µM SM, *p* < 0.01 vs. H_2_O_2_ alone; *n* = 4; expression of p53, 0.3 and 1 µM SM, *p* < 0.01; 3 µM SM, *p* < 0.001 vs. H_2_O_2_ alone; *n* = 4). The effect of SM on cleaved caspase-3 (CC3) expression was also determined by Western blotting. The H_2_O_2_ treatment led to a significant increase in the CC3 level (*p* < 0.001, *n* = 4) in HAECs, and this effect was inhibited by 3 μM SM (*p* < 0.001, *n* = 4) (Figure 5A). We further evaluated whether the changes in p53 and CC3 expression were mediated by IKKα. We treated HAECs with si-IKKα and si-Ctl (control), and the expression levels of P-IKKα, p53, and CC3 were assessed by Western blotting. As shown in Figure 5B, the levels of P-IKKα, p53, and CC3 significantly increased in H_2_O_2_-treated HAECs (*p* < 0.001, *n* = 4); however, the levels of P-IKKα, p53, and CC3 decreased following pre-treatment of H_2_O_2_-treated HAECs with si-IKKα (P-IKKα and CC3, *p* < 0.001 and p53, *p* < 0.05 vs. H_2_O_2_-treated alone; *n* = 4, Figure 5B). Additionally, in vivo quantitative real-time PCR analysis showed that the mRNA level of *IKKα*, *p53*, and *caspase 3* or Western blotting data showed protein levels of P-IKKα, p53, and CC3 were significantly increased in the carotid artery of 5/6 Nx ApoE^–/–^ mice compared with sham group (Figure 5C–F), whereas they were decreased in the carotid artery of 5/6 Nx ApoE^–/–^ mice pretreated with SM (Figure 5C–F). These results indicated that H_2_O_2_ induced apoptosis via the P-IKKα/p53/CC3 pathway.

## 4. Discussion

In the present study, we demonstrated that 5/6 Nx ApoE^–/–^ mice showed significantly increased levels of MDA in the kidney, plasma, and carotid artery, indicating lipid peroxidation, and increased numbers of atherosclerotic lesions in the aorta and carotid artery, as assessed by Oil Red O staining and hematoxylin and eosin staining, respectively. Furthermore, these effects were significantly attenuated by SM treatment. SM could also suppress H_2_O_2_-induced oxidative stress and injury in HAECs. Moreover, the data suggested that this may be mediated by a reduction in the level of P-IKKα and an inhibition of the p53/caspase-3 pathway in HAECs. These findings indicate that SM improved ROS-induced endothelial cell damage and atherosclerotic CVD due to renal injury. Thus, renal injury potentiated atherosclerosis, which is consistent with clinical observations and past experimental evidence [22].

The increased risk of premature death in patients with chronic renal failure is associated with the systemic, chronic pro-inflammatory state in CKD, which contributes to a remodeling of the blood vessels and the myocardium, leading to the formation of atherosclerotic lesions [1]. A suggested link between CKD and CVD is endothelial dysfunction and oxidative stress [22]. The consequences of oxidative stress in patients with CKD include atherosclerosis, protein denaturation, and a shortened red blood cell life span. An increase in ROS levels possibly triggers the remodeling of blood vessels and myocardium, leading to the formation of atherosclerotic lesions [3]. Therefore, studying the mechanism of ROS generation in CKD-mediated atherosclerosis and the potential interventions to protect endothelial cells from ROS-induced injury can be beneficial to prevent CVD [2,23].

SM has a vital role in diminishing endothelial oxidative stress [24]. SM reduces ROS generation in human neuronal cells exposed to H_2_O_2_ [13]. In the present study, SM inhibited ROS production induced by H_2_O_2_ in HAECs. These results indicate that SM exerted a protective effect by inhibiting H_2_O_2_-induced cell injury. High concentrations of SM are cytotoxic to hepatocellular carcinoma cells [12]. Our findings showed that SM has a protective effect at all tested concentrations (0.3, 1, and 3 μM), and that it had no effect on the viability of HAECs, which suggested that SM exerted beneficial effects on the endothelium without any negative adverse effects. ROS are produced during normal cellular metabolism [25]. When there is an imbalance between the levels of free radicals generated and antioxidant defense systems, excessive ROS is generated, which can damage proteins and DNA, leading to endothelial dysfunction [23]. This contributes to the progression of cerebral and vascular diseases [2,4,8]. H_2_O_2_ plays a considerable role in vascular and endothelial dysfunction [23]. High levels of H_2_O_2_ can induce significant injury and diminish endothelial cell viability [6,23]. Our results also showed that H_2_O_2_ significantly reduced cell viability and resulted in DNA damage in HAECs. However, pre-incubation of the cells with SM 1 h before the addition of H_2_O_2_ significantly suppressed H_2_O_2_-induced cell death. These results demonstrate the anti-apoptotic property of SM.

The ternary IKK complex comprises IKKα, IKKβ, and IKKγ (NEMO) [26], and signaling through this complex induces the activation of nuclear factor kappa-B (NF-κB), involved in cancer, immune responses, inflammation, and cell survival [26,27]. IKKα is stimulated by H_2_O_2_ [28]. Additionally, IKKα plays a key role in p53-induced cell apoptosis [29]. To elucidate the mechanisms involved in the protection of endothelial cells by SM against oxidative stress injury, we investigated the effects of SM on IKKα and p53 activation in HAECs exposed to H_2_O_2_. IKKα mediated the stimulation of p53 activation in HAECs treated with H_2_O_2_. These results suggest that IKKα plays a supportive role in ROS-mediated apoptosis by modulating p53 transcriptional activity.

Caspase-3 is an effective indicator of the degree of cellular apoptosis [30]. Caspase-3 is mainly activated via chromatin condensation and DNA fragmentation in the nucleus [30]. Caspase-3 is the main protease involved in the apoptosis of endothelial cells as it functions downstream of other caspases and is involved in initiating the apoptotic cascade [11,30]. In this study, caspase-3 activation was increased by H_2_O_2_ in HAECs, and this could be prevented by pretreating cells with an siRNA targeting IKKα. Furthermore, the in vivo mRNA expression of IKKα, p53, and caspase-3, or the protein expression of P-IKKα, p53, and cleaved caspase-3 decreased in the carotid artery tissues of 5/6 Nx ApoE^–/–^ mice following SM treatment, similar to those observed in in vitro experiments. Based on these findings, we hypothesize that H_2_O_2_-induced apoptosis and atherosclerosis occurs via the IKKα/p53/caspase-3 pathway and that SM can inhibit the activity of this pathway.

As mentioned earlier, the ROS generated in CKD can oxidize biomolecules such as lipids, which further increases cell toxicity, kidney injury, and atherosclerosis [4,31]. Additionally, sesamolin, a major constituent of sesame oil, reportedly inhibits lipid peroxidation in rat livers and kidneys [32]. Our results showed that in 5/6 Nx ApoE^–/–^ mice, SM protected the endothelial cells and arrested atherosclerosis to attenuate ROS-injury arising as a result of chronic kidney injury. SM administration reportedly improves ferric–nitrilotriacetate-induced acute kidney injury in mice, which supports our findings [17]. In addition, kidney damage caused by other factors, including glomerular lipidosis—associated with dyslipidemic conditions prone to the development of kidney disease—or a dramatic increase in immune/inflammatory responses can worsen atherosclerosis and impact the occurrence of atherosclerotic artery disease [33,34]. In our preliminary data, kidney hematoxylin and eosin staining revealed that the 5/6 Nx group had increased glomerular lipidosis, which can lead to decreased kidney function [33]. Meanwhile, glomerular lipidosis was decreased in the 5/6 Nx mice treated with SM; within this group, SM was found to protect against ROS injury and ameliorate ROS-induced glomeruli damage. Moreover, in our preliminary data, in addition to improving the direct endothelium impact in the nephropathy 5/6 mouse model, SM has beneficial effects on kidney function while improving traditional risk factors related to arteriosclerosis. In addition, SM appeared to improve the inflammation caused in the 5/6 nephropathy model. Hence, SM may represent a promising treatment for kidney disease.

ROS from other sources, including advanced glycation end products (AGEs) or mitochondrial-derived ROS can also contribute to endothelial cell damage and CVD [6,8,23,35,36]. In the present study, we exclusively focused on ROS generation in response to exogenous H_2_O_2_; hence, further investigation into AGEs and mitochondrial ROS are needed to elucidate their role in cell damage. Additionally, although antioxidant strategies have potential, only a few interventional studies have examined their effects. Hence, large, randomized, long-term studies are required to establish the efficacy and safety of SM in patients with CKD.

## 5. Conclusions

Overall, SM could inhibit H_2_O_2_-induced endothelial cell injury by direct inhibition of intracellular ROS generation. These protective effects were closely associated with the suppression of IKKα phosphorylation and inhibition of the apoptotic death cascade through the suppression of p53 and a reduction in caspase-3 activation. Additionally, SM also improved CKD-related outcomes in the 5/6 Nx mouse model of CKD-induced atherosclerosis. The findings provide an important basis to better understand the action of SM on HAECs and the potential beneficial effects of SM treatment in CKD and CVD.

## Figures and Tables

**Table 1:** List of primers used in qPCR.

Gene Expression qPCR Primers		
*GAPDH*	5′-AGAAGGCTGGGGCTCATTTG-3′	5′-AGGGGCCATCCACAGTCTTC-3′
*p53*	5′-TACAAGAAGTCACAGCACAT-3′	5′-GATAGGTCGGCGGTTCAT-3′
*IKKα*	5′-GCCAGGGAGACTTGATGG-3′	5′-GAGGTCTGTGCTTTAGCTGCTT-3′
*Caspase-3*	5′-GCGATGGAGAATGTGCATAAATTC-3′	5′-GGGAAACCAACAGTACTCAGTCCT-3′

## Data Availability

The data presented in this study are available in article.
